# Detection of coronary lesions in Kawasaki disease by Scaled-YOLOv4 with HarDNet backbone

**DOI:** 10.3389/fcvm.2022.1000374

**Published:** 2023-01-20

**Authors:** Ho-Chang Kuo, Shih-Hsin Chen, Yi-Hui Chen, Yu-Chi Lin, Chih-Yung Chang, Yun-Cheng Wu, Tzai-Der Wang, Ling-Sai Chang, I-Hsin Tai, Kai-Sheng Hsieh

**Affiliations:** ^1^Department of Pediatrics, Kawasaki Disease Center, Kaohsiung Chang Gung Memorial Hospital, College of Medicine, Chang Gung University, Kaohsiung, Taiwan; ^2^Department of Computer Science and Information Engineering, Tamkang University, New Taipei City, Taiwan; ^3^Department of Information Management, Chang Gung University, Kaohsiung, Taiwan; ^4^Department of E-Sport Technology Management, Cheng Shiu University, Kaohsiung, Taiwan; ^5^Department of Medicine, College of Medicine, China Medical University, Taichung, Taiwan; ^6^Department of Pediatric Cardiology, China Medical University Children's Hospital, China Medical University, Taichung, Taiwan; ^7^Center of Structure and Congenital Heart Disease/Ultrasound and Department of Cardiology, Children's Hospital, China Medical University, Taichung, Taiwan

**Keywords:** Kawasaki disease, echocardiography, deep learning, object detection, Scaled-YOLOv4, HarDNet, coronary dilatation and brightness

## Abstract

**Introduction:**

Kawasaki disease (KD) may increase the risk of myocardial infarction or sudden death. In children, delayed KD diagnosis and treatment can increase coronary lesions (CLs) incidence by 25% and mortality by approximately 1%. This study focuses on the use of deep learning algorithm-based KD detection from cardiac ultrasound images.

**Methods:**

Specifically, object detection for the identification of coronary artery dilatation and brightness of left and right coronary artery is proposed and different AI algorithms were compared. In infants and young children, a dilated coronary artery is only 1-2 mm in diameter than a normal one, and its ultrasound images demonstrate a large amount of noise background-this can be a considerable challenge for image recognition. This study proposes a framework, named Scaled-YOLOv4-HarDNet, integrating the recent Scaled-YOLOv4 but with the CSPDarkNet backbone replaced by the CSPHarDNet framework.

**Results:**

The experimental result demonstrated that the mean average precision (mAP) of Scaled-YOLOv4-HarDNet was 72.63%, higher than that of Scaled YOLOv4 and YOLOv5 (70.05% and 69.79% respectively). In addition, it could detect small objects significantly better than Scaled-YOLOv4 and YOLOv5.

**Conclusions:**

Scaled-YOLOv4-HarDNet may aid physicians in detecting KD and determining the treatment approach. Because relatively few artificial intelligence solutions about images for KD detection have been reported thus far, this paper is expected to make a substantial academic and clinical contribution.

## 1. Introduction

Kawasaki disease (KD), a systemic vasculitis predominantly affecting medium-sized arteries (1, 2). It may accelerate coronary arteriosclerosis and sudden death risks, and it is the leading cause of acquired heart disease in children of many developed countries (3). The global incidence of Kawasaki Disease (KD) has increased in the last 10–20 years, with an annual incidence rates per 100,000 children <5 years old around 100–300 in Japan and northern Asia regions (4). It is therefore a significant disease burden for children.

KD-associated coronary lesions (CLs) are the most serious cardiovascular sequelae and can lead to acute intra-coronary thrombosis and stenosis (5). These lethal complications often occur in KD patients with delayed use of intravaenous immnunosglssosbulin (IVIG) or refractory to initial IVIG (6). Abou Sherif et al. (7) demonstrated the effects of coronary artery aneurysms on human health. Therefore, timely assessment of patients with KD using imaging modalities is the key factor ensuring the effective lowering of long-term cardiovascular events in KD patients.

Among multi-modality imaging used to evaluate KD-associated CLs, echocardiography is the most common to identify patients with KD as it is non-invasive, easy-accessible, widely available, and cost-effective and provides a real-time qualitative assessment of the coronary system (8). At present time, echocardiography has to be assessed by cardiologist. The echocardiography contains large amount of information. It is therefore logic to employ the advanced bioinformatics technology to assess the echocardiography in patients with KD (9, 10).

Artificial intelligence (AI), deep learning (DL), is basically a number of neural network-related algorithms to identify patterns with purpose and has been widely applied in the medical field to assist in diagnosis. Its power comes from its ability to find these associations from large amounts of data and draw non-linear relationships between various predictors and an outcome of interest without background knowledge. There are numerous applications of deep learning in cardiac ultrasound image classification (11–14) described briefly in Table 1. In addition, some important object detection algorithms belonged to the category of DL were recently proposed (21–23) organized in Table 2. However, to the best of our knowledge, there are no relevant studies regarding applied AI for CLs assessment in patients with KD.

**Table 1:** Summary of cardiac medical image researches.

**References**	**Problem**	**Method**
Gao et al. (11)	Cardiac classification	Optical flow technology and CNN
Nascimento and Carneiro (15)	Segmentation	Manifold learning with DBNs
Chen et al. (12)	Ultrasound image classification	Fully convolutional network and transfer learning
Bridge et al. (16)	Enhance ultrasound images and localize fetal heart disease	Bayesian inference and regression forest
Poudel et al. (17)	CT image classification	Recursive full CNN
Avendi et al. (18)	Left ventricle segmentation	CNN andstacked autoencoder
Wolterink et al. (19)	Coronary artery calcification quantization in cardiac CT	Paired CNNs
Gungor et al. (13)	View Classification and Object Detection in Cardiac Ultrasound	InceptionV3 and Faster-RCNN with ResNet101
Chen et al. (14)	Object detection on the ventricular septal defects of doppler ultrasound images	YOLOv4-DenseNet
Sirjani et al. (20)	Ventricle segmentation	EchoRCNN for deep video object segmentation

**Table 2:** Major characteristics of the object detection algorithms.

**References**	**Name**	**Method**
Redmon et al. (24)	YOLO	The first one-stage approach
Liu et al. (25)	SSD	One-stage approach inspired by YOLO
Redmon and Farhadi (26)	YOLOv2	K-means algorithm generates anchors, Darknet-19 as the backbone, Wordtree, and batch normalization
Lin et al. (27)	RetinaNet	CNN model with a Feature Pyramid Network
Redmon and Farhad (28)	YOLOv3	ResNet as the backbone, upsampling, FPN, and three scales of output heads
Tian et al. (29)	YOLOv3-DenseNet	Replace the ResNet by DenseNet as the backbone
Bochkovskiy et al. (21)	YOLOv4	CSP, SPP, FPN, PAN, mosaic augmentation, label smoothing, mish function, and so on
Jocher (22)	YOLOv5	CSPized the neck and a focus layer
Wang et al. (23)	Scaled-YOLOv4	Different scales for various running environments

## 2. Method

**Figure 1:**
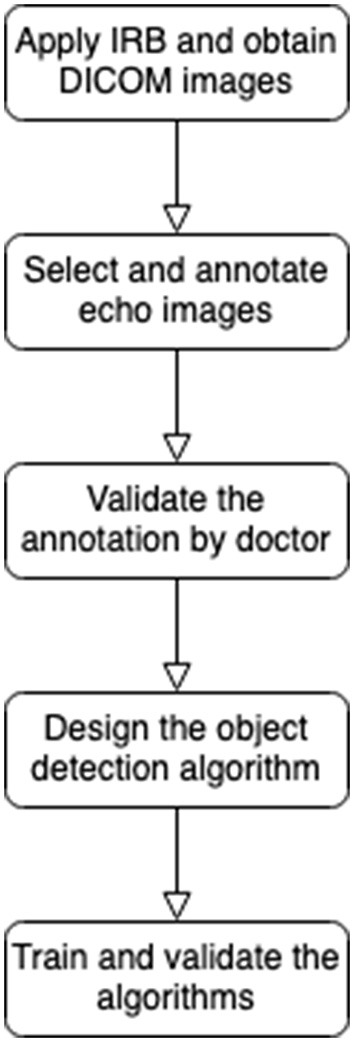
FlowChart of this research.

There are five major procedures of this study depicted in Figure 1. First of all, this retrospective study included cardiac ultrasound images from patients with KD in Kaohsiung Chang Gung Hospital from June 1, 2000, to June 30, 2020 (IRB approving No. 202001238B0C502). The extracted data were patient age, body height, body weight, cardiovascular diameter of coronary arteries, and cardiac ultrasound images in the DICOM format. The inclusion criterion was the receipt of KD diagnosis (International Classification of Diseases, Ninth Revision, Clinical Modification code: 446.1, or International Classification of Diseases, Tenth Revision code: M303).

All the echocardiography was performed by board-certificated pediatric cardiologists. The echocardiographic images of coronary arteries were obtained according to the standard procedure as shown in the scientific statement of American Heart Association on Kawasaki Disease (10). The inter-observer variability was around 0.90–0.95 by laboratory quality check.

After anonymization of the obtained echo image data. As such, the privacy of the included patients was not infringed upon. We labeled region of interest on the images using LabelMe. The annotation results was validated by another experienced cardiologist. Considering the retrospective nature of this study, we did not consider additional behavioral factors of the included children. Thereafter, in-depth learning was performed using different models and evaluate which object detection algorithm will perform better.

### 2.1. Research definition

**Figure 2:**
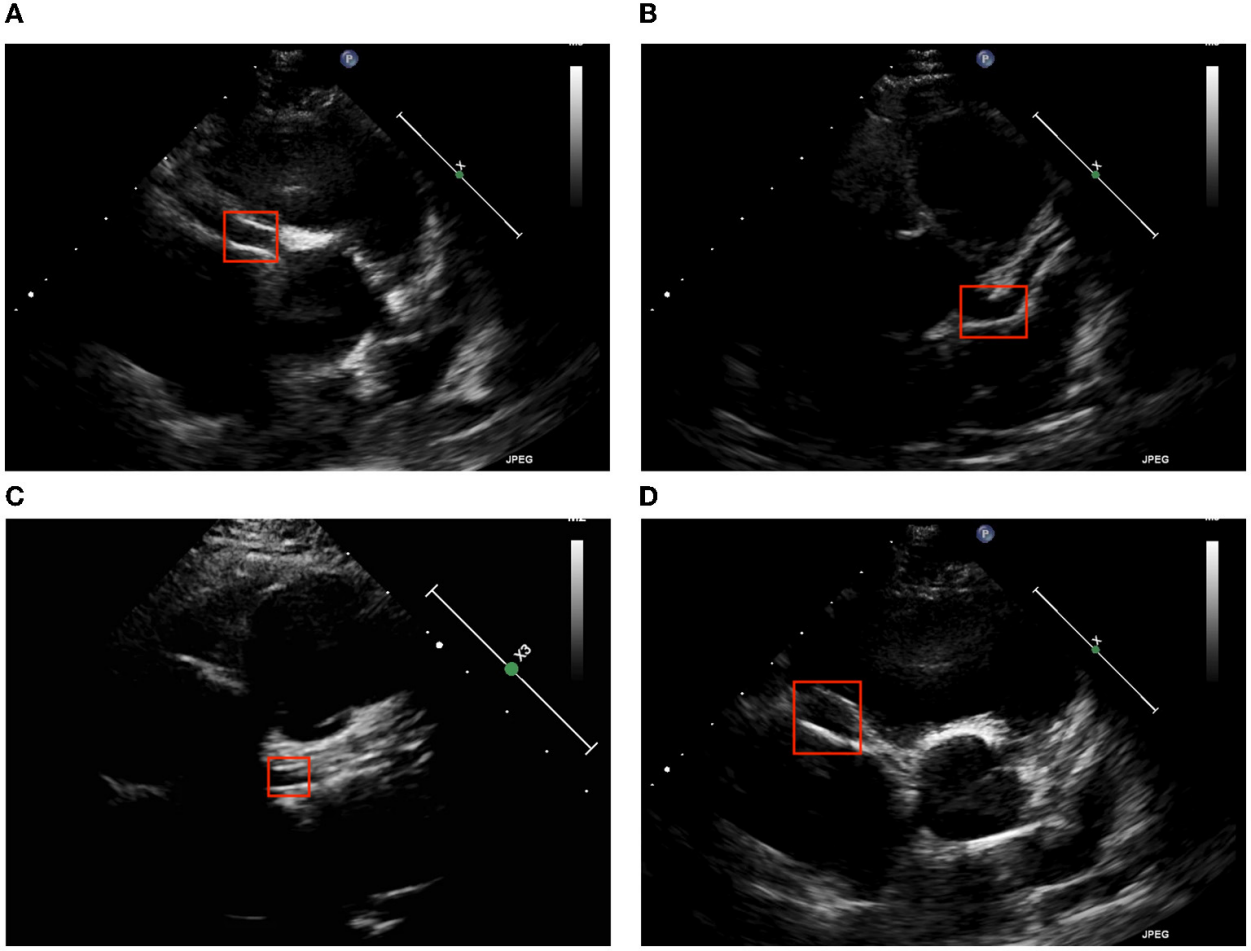
Two major echocardiographic coronary patterns in children with KD. **(A)** LCA brightness, **(B)** LCA dilatation, **(C)** RCA brightness, and **(D)** RCA dilatation.

Coronary arteries can be divided into left coronary arteries (LCAs) and right coronary arteries (RCAs). One of the main symptoms of KD is CLs, which are detectable on cardiac ultrasound. Clinically, short axis ultrasound can be used to assess for abnormalities in LCAs and RCAs. Figure 2 presents ultrasound images from Kaohsiung Chang Gung Hospital; we took two features of CLs, brightness and dilatation (10), to be detected.

**Figure 3:**
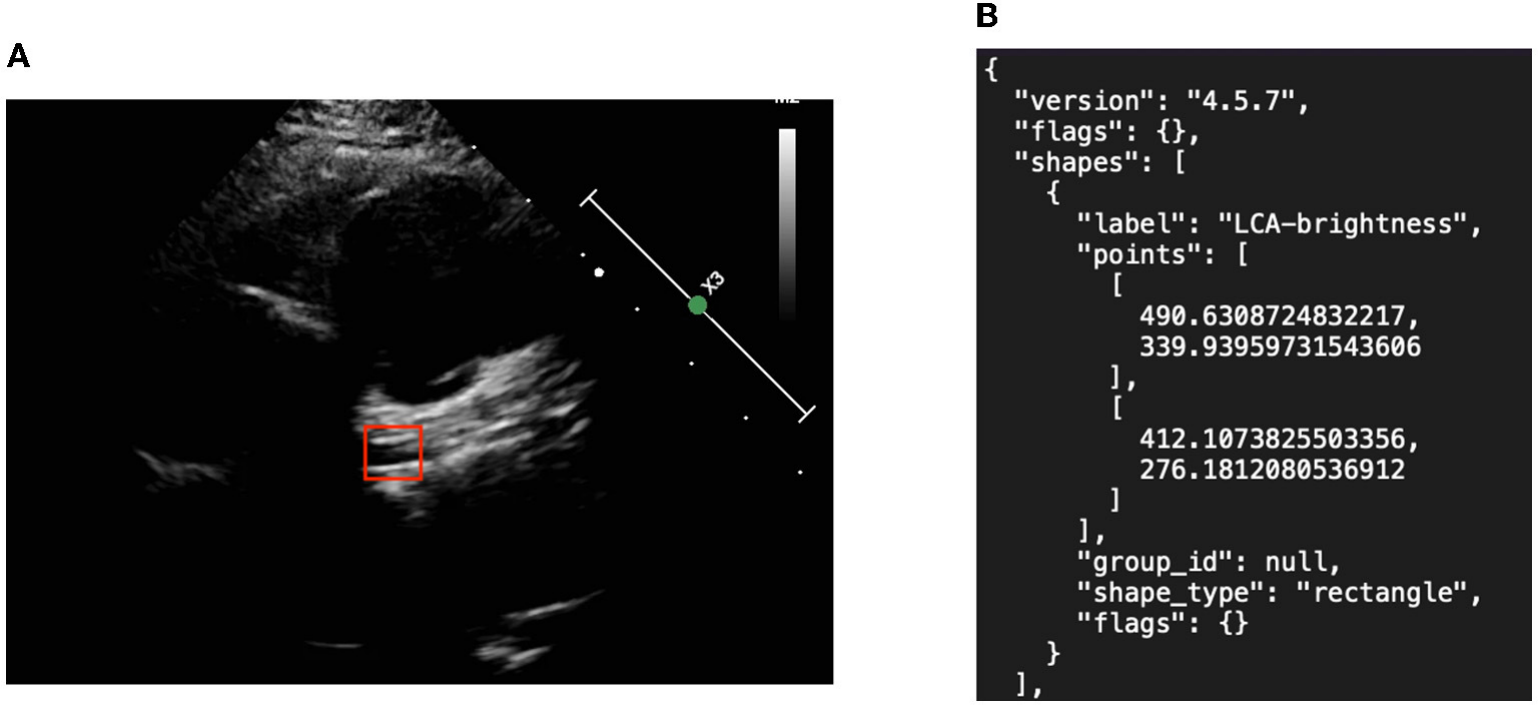
Object detection picture and annotation format in JSON. **(A)** Left coronary artery annotation and **(B)** JSON file.

We created object detection algorithms to learn the precise location of the two major symptoms. Image annotation was required to mark the location of the aneurysm as shown in Figure 3. Subsequent algorithms were designed to read the contents of the JSON materials, including file location, disease attribute, and corresponding location. In addition, the association between data amplification method and marking results, such as the image rotation-marking frame relationship, was considered so as to maximize the advantages of data amplification.

### 2.2. Scaled-YOLOv4-HarDNet object detection algorithm

**Figure 4:**
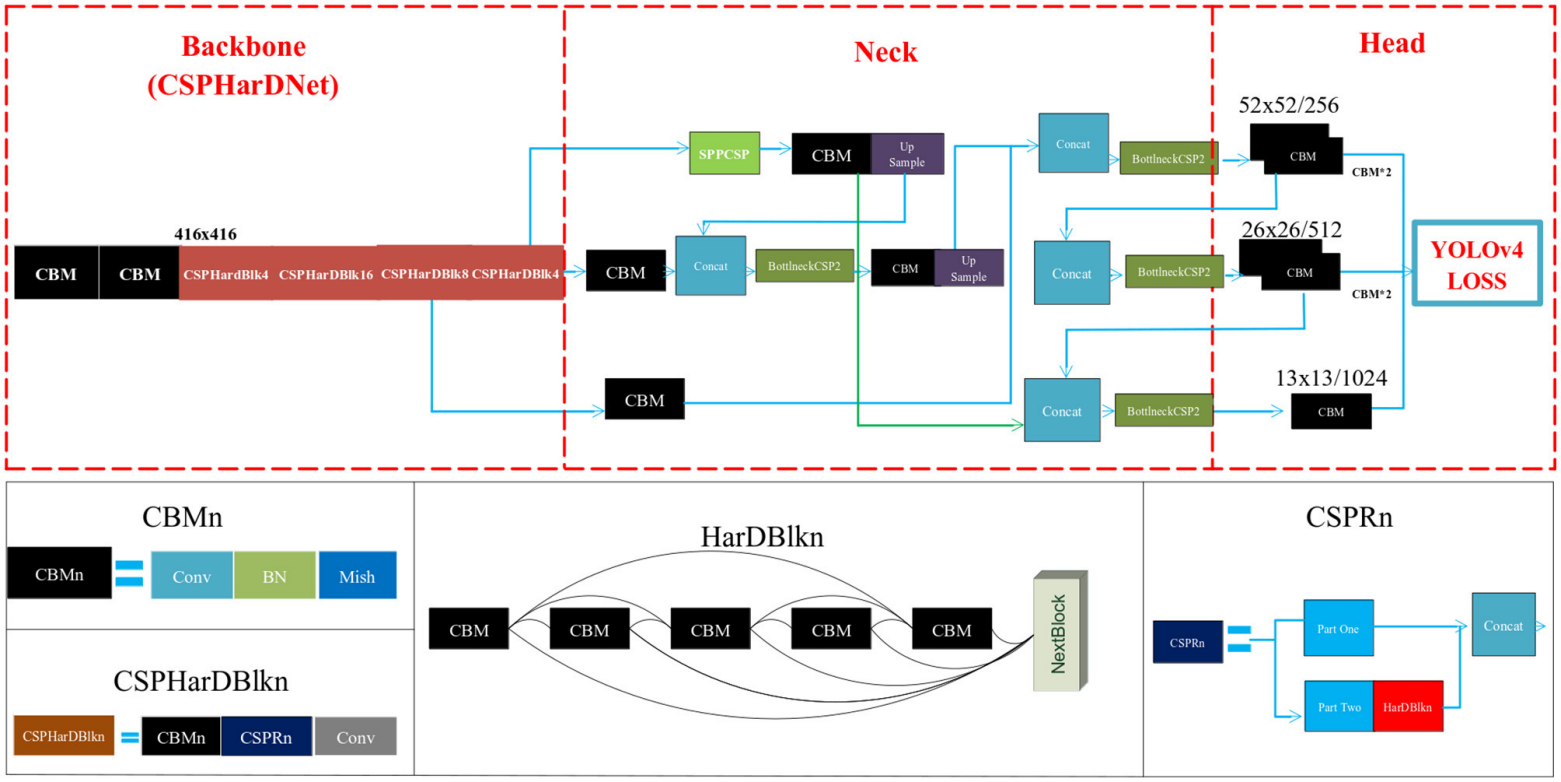
Scaled-YOLOv4-HarDNet framework.

The main framework of Scaled-YOLOv4-HarDNet is depicted in Figure 4. Because the proposed algorithm adopts the main features of Scaled-YOLOv4 and HarDNet, we illustrate the original settings and also explain the key modification items of Scaled-YOLOv4. Scaled-YOLOv4 integrates various methods, such as the cross stage partial network (CSPNet), spatial pyramid pooling with CSP (SPPCSP), FPN, path aggregation network (PANet), mish activation function, label smoothing, and complete IOU (CIoU) loss function.

The use of CSPHarDNet as the backbone is one of the main features of the proposed algorithm, and the input of CSPHarDNet is CBM, which is formed through a combination of convolution layers and the batch normalization and Mish activation function. The input resolution of the first convolution layer is 416 × 416. The Mish function is a self-regularized nonmonotonic neural activation function, which increases the penetration of information in the neural network. Moreover, ZCRn comprises zero pool, CBM and CSPRn. CSPRn refers to the CSPNet framework, with *n* denoting the number of copies. CSPNet divides the function scheme into two parts: In the first part, gradient change is retained in the feature map from the beginning to the end to ensure the accuracy and reduce the amount of calculation and storage cost required. The second part involves the skip connection approach of ResNet. The first part is consistent with the feature map of the second part. The output resolutions of three output heads are 52 × 52, 26 × 26, and 13 × 13, respectively.

Scaled-YOLOv4 uses FPN and PANet methods in the Neck area, whereas YOLOv3 only uses FPN. FPN performs upsampling from smaller to larger resolutions and then connects with the larger ZCRn. The PANet framework applies bottom-up path enhancement to the previous local convolution layer by performing the upsampling operation. Its advantage includes shortening the information path between high- and low-resolution features.

Originating from YOLOv5 (22), Scaled-YOLOv4 adopts CSPNet in Neck to increase the training and detection speed further. The activation function in Neck can be replaced by the Mish function. Scaled-YOLOv4 is applicable in different environment based on depth requirements. For instance, the P5 architecture can be adopted in an edge computing environment, and the P6 architecture can be adopted in general environments. For larger GPU devices, such as the Tesla V100, the P7 architecture can be used for training and prediction. Thus, we compared our algorithm with YOLOv5 by using the P5 architecture.

Scaled-YOLOv4 uses a head area identical to that of YOLOv3 and outputs the same three dimensions with 76 × 76 (256 feature maps), 38 × 38 (512 feature maps), and 19 × 19 (1,024 feature maps) resolution when the input resolution is 608. When the resolution is reduced to 416 × 416, the output resolution of the three dimensions are 13 × 13 (1,024 feature maps), 26 × 26 (512 feature maps), and 52 × 52 (256 feature maps). The only change in the head area is the loss function, and the loss function for Scaled-YOLOv4 is CIoU, which measures the difference between the real and the prediction boxes.

Finally, in our proposed algorithm Scaled-YOLOv4-HarDNet, there are four variants which are the combination of the activation function and the number of layers. Firstly, we substitute the original activation function ReLU and to be Mish function. Secondly, because the number of layer in a deep learning algorithm also influences the performance, we select the number of layers in the CSPHarDNet includes 39 and 68. Instead of using the full name of Scaled-YOLOv4-HarDNet, the four variants are named HarDNet39-ReLU, HarDNet39-Mish, HarDNet68-ReLU, and HarDNet68-Mish in short. This study will conduct extensive experiments and compare the proposed algorithms with two existing benchmark algorithms.

## 3. Experimental results

In total, 1,283 images–of which 395 and 472 were on the LCA for brightness and dilatation, respectively; 174 and 242 were on the RCA for brightness and dilatation, respectively–were included in the validation dataset. The number of images varied due to the coronary artery symptoms of KD tending to occur in the LCA and RCA. After image arrangement, we use 70% of images to train our proposed algorithms, 20% of images are as the validation dataset during the training, and the rest of 10% of images as the test dataset. Two well-known benchmark algorithms, Scaled-YOLOv4 and YOLOv5, are applied into the comparison. The following training parameters: batch = 72 and epoch = 300 are used across the experiments. To carry out these experiments, we employed the PyTorch environment with Nvidia NGC container 21.08 on a Tesla V100 server of Taiwan Computing Cloud.

After each algorithm was trained, we used the rest of 10% of images to test the real performance of these algorithm. The test results are presented in Table 3. We list the performance of each algorithm on the four patterns of LCA and RCA via the mean average precision (mAP) metric in percentage. The best mAP value of each class is shown in bold. The overall column shows the average results of the four patterns for each compared algorithm. Among them, HarDNet68-Mish achieves the highest overall mAP score to be 72.63% and the HarDNet39-ReLU (71.08%) is the inferior version of our proposed method. However, HarDNet39-ReLU is still better than Scaled-YOLOv4 (70.05%) and YOLOv5 (69.79%). The models including HarDNet had significantly improved outcomes compared with the original Scaled YOLOv4; moreover, Mish function also led to a better performance than ReLU.

**Table 3:** Comparison among models on the four patterns via mAP metric (%).

**Algorithm**	**LCA brightness**	**LCA dilatation**	**RCA brightness**	**RCA dilatation**	**Overall**
Scaled-YOLOv4	61.37	82.98	64.98	70.86	70.05
YOLOv5	58.98	84.85	61.86	73.45	69.79
HarDNet39-ReLU	60.07	83.42	67.03	71.58	70.52
HarDNet39-Mish	63.77	81.42	65.3	**73.65**	71.04
HarDNet68-ReLU	**66.5**	81.86	66.73	69.24	71.08
HarDNet68-Mish	63.85	**85.41**	**74.22**	67.04	**72.63**
Average	62.43	83.32	66.69	70.97	70.85

The best mAP value of each class is shown in bold.

From the four image types, the average result of LCA dilatation is >83.32% which shows the LCA dilatation is easier to be detected. However, RCA dilatation is not as high as the one of LCA dilatation. Moreover, the pattern of LCA brightness has the lowest mAP in average which implies LCA brightness is the most difficult one to be detected.

The effectiveness of the aforementioned methods was compared using different Intersections over Union (IoU) as criteria; the comparison of AP50, APsmall, APmedium, ARsmall, and ARmedium is presented in Table 4. Here, AP represents the average precision or accuracy, AR the average recall, and AP50 the AP with IoU = 50% and “small” and “medium” represent the sizes of the bounding boxes; we did not include a large classification because no large-sized objects were considered. The best AP value of each class is also shown in bold. The result of the AP50 is quite similar to the one of mAP-50 because HarDNet68-Mish is best algorithm which outperform the Scaled-YOLOv4 and YOLOv5. When it comes to the metrics of APsmall or ARsmall, HarDNet68-Mish is also the best one compared with others. However, if we apply the metrics of APmedium and ARmedium, Scaled-YOLOv4 is better than the proposed algorithms.

**Table 4:** AP and AR results of the compared algorithms.

**Algorithm**	**AP50**	**APsmall**	**APmedium**	**ARsmall**	**ARmedium**
Scaled-YOLOv4	70.1	28.54	**40.55**	30.83	**51.36**
YOLOv5	69.81	30.46	37.56	32.64	46.33
HarDNet39-ReLU	70.51	29.64	39.67	32.92	50.7
HarDNet39-Mish	70.55	30.33	37.16	32.08	49.32
HarDNet68-ReLU	70.81	32.09	39.14	33.61	50.41
HarDNet68-Mish	**72.57**	**33.34**	39.93	**35.42**	50.7

The best AP value of each class is also shown in bold.

## 4. Discussion

This pioneer, pilot study has demonstrated that several AI based algorithms showed encouraging result. The assessment of coronary morphology could be performed by AI is promising and showed that assessment of coronary morphology may be assisted by AI algorithm in the future and may thus greatly decrease the work-loading by cardiologist using conventional assessment of coronary morphology for echocardiography in patients with KD.

In the proposed Scaled-YOLOv4-HarDNet, we verified the 39 and 68 HarDNet layers and substituted the ReLU function with the Mish function. We the impact of the number of layers improves the solution quality which is expected. However, it does not guarantee to yield better prediction quality when it comes to 85 layers in our pilot experiments. Then, Mish function generally enhances the performance when it is compared to the ReLU function compared to its original setting. It is noticeable that the performance of HarDNet39-Mish is almost equal to the one of HarDNet68-ReLU. As a result, if the object detection model is designed to be installed on an edge device, we could consider the HarDNet39-Mish because the model size is smaller.

When we compared the proposed algorithm with Scaled-YOLOv4 and YOLOv5, even though HarDNet39-ReLU is the inferior version of the proposed method, it is better than the two benchmark algorithms. Then, HarDNet68-Mish yielded the optimal overall mAP (72.63%) across the four features compared with Scaled-YOLOv4 and YOLOv5 (70.05 and 69.79%, respectively).

The four patterns also have different difficulty levels. LCA dilatation has the better detection result from no matter which object detection algorithm is applied. However, the performance of the brightness pattern of LCA and RCA is poor. The primary reason is that the characteristic of dilatation shows that the color inside the vessel is black and white outside the vessel. However, the brightness pattern is the grayish white of the vessel wall compared to the grayish color in the background. As a result, the brightness pattern is harder to detect.

COCO metrics include average precision and average recall for bounding boxes of different sizes. Here, the proposed Scaled-YOLOv4-HarDNet, with 68 layers and the Mish function, significantly outperformed Scaled-YOLOv4 and YOLOv5 in terms of the average precision and average recall for small bounding boxes. However, for the medium-sized bounding box, Scaled-YOLOv4, but not YOLOv5, performed <0.66% better than did the proposed framework. Thus, our COCO metrics results indicated that proposed algorithm can detect small objects extremely well and medium-sized objects adequately. Finally, because we did not encounter any large objects in the medical images in this study, how the performance of our proposed algorithm for such objects compares with the performance of the two benchmark algorithms remains unknown.

According to these results, the strength of the proposed algorithms are to detect the two KD patterns on echocardiographic images, and also perform objective assistance for cardiologist. Finally, varies of echo equipment models, ultrasound resolution, case numbers, and different results from different cardiovascular specialist in different time may be the limitation of this study. Due to the medical images are available from the Philip EPIQ 7C and iE33 belonged to the studied hospital, it limits the capability of reading the echo images of other manufacturer due to the color and resolution, frame rate are different. On the other hand, when there are more cardiovascular specialists are involved in the research, it is beneficial to validate the labeling results in many rounds and yield results precisely. The results from this study still need further validation from different researches before a conclusion.

## 5. Conclusions

KD is the leading cause of acquired heart disease in children especial in Asia. Precisely diagnosis and treatment is the major point to prevent coronary lesions in KD. We proposed the Scaled-YOLOv4-HarDNet, which integrates HarDNet as the backbone rather than DarkNet. In the four variants of the proposed algorithm with different number of layers and activation functions, they are better than the two benchmark algorithms Scaled-YOLOv4 and YOLOv5. Moreover, the proposed algorithm is good at the smaller object size of coronary artery in children which is suitable to detect the lesion in LCA and RCA.

The results from this study will help clinician to identify coronary artery lesion objectively from the help of AI and further support the detection of KD.

## Data availability statement

The raw data supporting the conclusions of this article will be made available by the authors, without undue reservation.

## Author contributions

H-CK provides the ultrasound images for investigation and supervised the study. S-HC is the paper's guarantor. Y-HC contributed to conception and design of the study. Y-CL annotated the medical images. Y-CW wrote the code and ran the experiments. C-YC and T-DW performed the statistical analysis. I-HT performed most echocardiography imaging in the investigation process, provided insight into coronary arteritis, and critically revised the manuscript. K-SH supervised image labeling corrections. All authors contributed to manuscript revision, read, and approved the submitted version.
